# Association of High Intakes of Vitamins B_6_ and B_12_ From Food and Supplements With Risk of Hip Fracture Among Postmenopausal Women in the Nurses’ Health Study

**DOI:** 10.1001/jamanetworkopen.2019.3591

**Published:** 2019-05-10

**Authors:** Haakon E. Meyer, Walter C. Willett, Teresa T. Fung, Kristin Holvik, Diane Feskanich

**Affiliations:** 1 Department of Community Medicine and Global Health, University of Oslo, Oslo, Norway; 2 Department of Nutrition, Harvard T.H. Chan School of Public Health, Boston, Massachusetts; 3 Division of Mental and Physical Health, Norwegian Institute of Public Health, Oslo, Norway; 4 Channing Division of Network Medicine, Department of Medicine, Brigham and Women’s Hospital, Harvard Medical School, Boston, Massachusetts; 5 Department of Nutrition, Simmons University, Boston, Massachusetts

## Abstract

**Importance:**

Vitamin supplementation far exceeding recommended doses is popular in segments of the population. However, adverse effects can occur. In a previous secondary analysis of combined data from 2 double-blind randomized clinical trials (RCTs), an unexpected increased risk of hip fracture was found among those treated with high doses of vitamin B_6_ in combination with vitamin B_12_.

**Objectives:**

To study if high intakes of vitamins B_6_ and B_12_ from food and supplements were associated with a risk of hip fracture in the Nurses’ Health Study and to investigate whether combined high intakes of both vitamins conferred a particularly increased fracture risk.

**Design, Setting, and Participants:**

In this prospective cohort study, 75 864 postmenopausal women in the United States were followed up from June 1984 through May 2014. The dates of analysis were July 2016 to June 2018. Information on hip fracture and a wide range of potential confounders was collected at baseline and with biennial follow-up questionnaires. Extensive dietary information was collected approximately every 4 years with a semiquantitative food frequency questionnaire. Relative risks (RRs) were calculated by Cox proportional hazards regression, with cumulative average intakes of vitamins B_6_ and B_12_ as main exposures, adjusting for potential confounders.

**Main Outcome and Measure:**

Hip fracture.

**Results:**

During follow-up, 2304 of 75 864 women had a hip fracture. Among the women with hip fractures, the median (range) age at hip fracture was 75.8 (46.7-93.0) years and the mean (SD) body mass index (calculated as weight in kilograms divided by height in meters squared) was 24.3 (4.6). Median (interquartile range) cumulative average intakes of total vitamins B_6_ and B_12_ were 3.6 (4.8) mg/d and 12.1 (11.7) μg/d, respectively. Both vitamin B_6_ (RR, 1.29; 95% CI, 1.04-1.59 for an intake of ≥35 vs <2 mg/d; *P* = .06 for linear trend) and vitamin B_12_ (RR, 1.25; 95% CI, 0.98-1.58 for an intake of ≥30 vs <5 μg/d; *P* = .02 for linear trend) were associated with increased fracture risk. Risk was highest in women with a combined high intake of both vitamins (B_6_ ≥35 mg/d and B_12_ ≥20 μg/d), exhibiting an almost 50% increased risk of hip fracture (RR, 1.47; 95% CI, 1.15-1.89) compared with women with a low intake of both vitamins (B_6_ <2 mg/d and B_12_ <10 μg/d).

**Conclusions and Relevance:**

In this cohort study, a combined high intake of vitamins B_6_ and B_12_ was associated with an increased risk of hip fracture. The intakes were far higher than the recommended dietary allowances. These findings add to previous studies suggesting that vitamin supplements should be used cautiously because adverse effects can occur.

## Key Points

**Question:**

Are high intakes of vitamins B_6_ and B_12_ associated with an increased risk of hip fracture?

**Findings:**

In a cohort study of 75 864 US postmenopausal women, 2304 had a hip fracture. A combined high intake of vitamins B_6_ and B_12_ was associated with an increased risk of hip fracture.

**Meaning:**

These results add to the evidence suggesting that caution should be used in vitamin supplementation when there is no apparent deficiency.

## Teaser

This cohort study assesses the association between high intakes of vitamins B_6_ and B_12_ from food and supplements with a risk of hip fracture in the Nurses’ Health Study and investigates whether combined high intakes of both vitamins were associated with an increased fracture risk.

## Introduction

Vitamin supplementation is widely popular. In the National Health and Nutrition Examination Survey (NHANES), approximately 50% of US adults reported taking at least 1 dietary supplement.^1^ The prevalence of use increased by age and was highest among individuals of non-Hispanic white race/ethnicity and among those with the highest education. Among US nurses, 28% reported using at least 4 dietary supplements.^2^

Both insufficient and excess intakes of a nutrient may be harmful. According to randomized clinical trials (RCTs), high-dose vitamin supplementation may lead to unexpected adverse effects.^3,4,5,6^ Based on the concept that high homocysteine concentrations could cause disease, several large RCTs have been performed. However, they failed to demonstrate a preventive effect of vitamin B supplementation on cardiovascular diseases and cancer,^7,8,9,10^ and possible adverse effects have been reported.^9,11,12^ Although hyperhomocysteinemia also may negatively influence bone quality by disturbing collagen cross-linkage and stimulating bone resorption,^13^ high doses of vitamin B_12_ and folic acid supplementation have not shown a fracture-preventing effect in RCTs.^14^ In a previous secondary analysis of combined data from 2 double-blind RCTs with factorial design,^15^ an unexpected increased risk of hip fracture was found among those treated with high doses of vitamin B_6_. The highest fracture risk was seen in the trial arm combining vitamin B_6_ and vitamin B_12_.

It is unlikely that new large trials of vitamin B_6_ and vitamin B_12_ will be carried out in the future. We aimed to study if high intakes of vitamins B_6_ and B_12_ from food and supplements were associated with a risk of hip fracture in the Nurses’ Health Study (NHS) and to investigate whether combined high intakes of both vitamins were associated with a particularly increased fracture risk.

## Methods

### Study Population

The NHS was initiated in 1976 among 121 701 female registered nurses in the United States aged 30 to 55 years. They responded to a mailed questionnaire concerning medical history, lifestyle, and disease risk factors. Every 2 years, follow-up questionnaires have been mailed to update individual characteristics and to identify incident diagnoses. Postmenopausal women entered the present analysis in 1984, when vitamin B supplement use was first assessed; otherwise, they entered the questionnaire cycle when they reached menopause. As shown in the flowchart in the eFigure in the Supplement, the final study sample included 75 864 postmenopausal women for our primary analysis. The women were followed up from June 1984 through May 2014. The dates of analysis were July 2016 to June 2018. The follow-up rate for this study population was 92% over the period of our analysis.

Completion and return of the self-administered questionnaires constituted informed consent. The investigation was approved by the Institutional Review Board at Brigham and Women’s Hospital, Boston, Massachusetts. This study followed the Strengthening the Reporting of Observational Studies in Epidemiology (STROBE) reporting guideline for cohort studies.

### Hip Fractures

On every biennial follow-up questionnaire, information about hip fracture (with the date of occurrence and a description of the circumstances) was requested. As nurses, they were expected to be capable of reporting hip fractures, as demonstrated in a small validation study^16^ in which all 30 self-reports were confirmed by medical records. Hip fractures were also identified from death records. For the primary cumulative average analysis, hip fractures were recorded in 2304 of 75 864 women during follow-up after exclusion of 117 fractures due to cancer or major traumatic events.

### Diet and Vitamin Supplement Use

Diet was assessed with a semiquantitative food frequency questionnaire (FFQ) in 1984, 1986, and every 4 years thereafter until 2010. Participants reported their habitual frequency of consumption over the previous year for specified serving sizes of more than 130 foods. Daily energy and nutrient intakes were calculated from the total diet.

The FFQ requested information on current use of supplements, such as vitamin B_6_, folic acid, vitamin B complex, vitamin B_12_–only supplements (beginning in 1998), multivitamins, vitamin A, vitamin D, and calcium. For vitamin B_6_ and vitamin A, information on daily dose was requested. For multivitamins, the respondents were asked to report the brand name and number of pills per week. Information on frequency and dose for other supplements was not obtained.

Vitamin intakes (except those from supplements only) were adjusted for total energy intake using regression analysis.^17^ In a validation study^18^ among 632 women in the NHS and the younger NHS2 cohort, correlations between intakes from the FFQ and 7-day diet records were 0.68 for total vitamin B_6_ and 0.68 for total vitamin B_12_. A correlation of 0.52 between total vitamin B_6_ in the food and plasma vitamin B_6_ (pyridoxal 5′-phosphate) and a corresponding correlation of 0.25 for vitamin B_12_ have been reported in another substudy within the NHS.^19^

### Nondietary Measures

On all biennial follow-up questionnaires, the following measures were assessed: weight; hours per week spent in recreational activities; smoking status; menopausal status and use of postmenopausal hormone therapy; diagnoses of cancer, diabetes, cardiovascular disease, and osteoporosis; and use of thiazide diuretics, furosemide-like diuretics, and oral corticosteroids. We calculated total metabolic energy expenditure (metabolic equivalent [MET] hours per week) from the reported recreational activities^20^ and body mass index (BMI) (calculated as weight in kilograms divided by height in meters squared) from current weight and height on the initial cohort questionnaire.

Questions concerning difficulties with balance, climbing a flight of stairs, or walking 1 block were included on questionnaires beginning in 1992, and questions on falls and pernicious anemia were included from 1998 onward. Self-rated general health status was requested in 1992, 1996, and 2000.

### Statistical Analysis

Participants were followed up to the date of first self-reported hip fracture or death from hip fracture, last questionnaire response, or the end of follow-up in 2014. In our primary analyses, we used vitamin B_6_, vitamin B_12_, and other nutrient intakes that were cumulatively averaged over follow-up. At the beginning of each new FFQ cycle, the intakes were updated with the mean of all assessments up to that time. Intakes were carried forward 1 cycle to replace missing data, and we excluded person-time in cycles in which women failed to report their dietary intake on the 2 most recent FFQs.

In alternate analyses, we used current intakes of vitamins and other nutrients (calculated as the mean of the 2 most recent FFQs) among 96 467 women with 2812 hip fractures (eFigure in the Supplement). Participants who had the 2 most recent dietary assessments missing for a cycle did not contribute person-time in that cycle.

Cross-sectional age-adjusted characteristics in the 2002 questionnaire cycle by categories of total vitamin B_6_ and B_12_ were recorded. We used Cox proportional hazards regression to compute hazard ratios (hereafter referred to as relative risks [RRs]) of hip fracture with 95% CIs according to predefined categories of vitamins B_6_ and B_12_. Cumulative average intakes of vitamins B_6_ and B_12_ were the main exposures. Separate analyses were performed for total vitamin intakes (from diet plus supplements) and intakes from supplements only. For vitamin B_6_, the 5 categories of total intakes ranged from less than 2 mg/d to at least 35 mg/d, and the 5 categories of intakes from supplements only ranged from 0 mg/d (no supplement use) to at least 25 mg/d. For vitamin B_12_, the 5 categories of total intakes ranged from less than 5 μg/d to at least 30 μg/d, and the 5 categories of intakes from supplements only ranged from 0 μg/d (no supplement use) to at least 25 μg/d. We also ran models with continuous intakes to test for linear trend. Based on the results from the previous RCT^15^ and from the categories used for vitamins B_6_ and B_12_, combined categories of vitamins B_6_ and B_12_ intakes were created.

All Cox proportional hazards regression models were stratified by age and questionnaire cycle to account for age and time. Relative risks were calculated from models using time-varying exposure and covariates (ie, person-time was assigned to the appropriate category for each variable at the beginning of every biennial follow-up questionnaire cycle). Multivariable RRs were computed from models that adjusted for potential dietary and nondietary confounding factors. For categorical covariates, missing data were assigned as a separate category. Less than 2% of the observations had missing data for BMI, physical activity, and smoking, and 5% of the observations had missing data for postmenopausal hormone therapy.

Multiplicative interactions between exposures were calculated using the Wald test for continuous data. The proportional hazards assumption was tested by including interaction terms between age and vitamin B_6_ and vitamin B_12_, respectively. In exploratory analyses, we also examined if the associations between vitamin B_6_ and vitamin B_12_ and risk of hip fracture differed by BMI and physical activity.

Data were analyzed using statistical software (SAS, version 9.4; SAS Institute Inc). Two-sided statistical tests were used, and *P* < .05 indicated statistical significance.

## Results

Among the 2304 fracture cases, the median age at hip fracture was 75.8 years (age range, 46.7-93.0 years). These women had a mean (SD) BMI of 24.3 (4.6), and median (IQR) cumulative average intakes of total vitamins B_6_ and B_12_ were 3.6 (4.8) mg/d and 12.1 (11.7) μg/d, respectively.

Height and BMI differed little across cumulative average intake categories of vitamin B_6_ (Table 1) and B_12_ (Table 2) in 2002, the approximate midpoint in follow-up, whereas physical activity increased and smoking prevalence decreased with higher intakes of both vitamins. Intakes of other micronutrients were also higher by increasing intakes of vitamins B_6_ and B_12_, whereas caffeine and alcohol consumption decreased. The reported prevalences of functional limitations, chronic diseases, and medication use tended to be lowest at low vitamin B_12_ intakes but were similar at middle and high intakes. Although less apparent, a similar pattern was found for vitamin B_6_. The Pearson product moment correlation coefficient between total intake of vitamins B_6_ and B_12_ was *r* = 0.51 (*P* < .001). Correlation coefficients between total vitamin B_6_ intake and the other vitamins were 0.35 for folate (*P* < .001), 0.28 for vitamin D (*P* < .001), and 0.37 for retinol (*P* < .001). For total vitamin B_12_ intake, the corresponding correlation coefficients were 0.43 for folate (*P* < .001), 0.33 for vitamin D (*P* < .001), and 0.36 for retinol (*P* < .001).

**Table 1.** Age and Age-Adjusted Characteristics of 61 445 Women in the Nurses’ Health Study Across Categories of Total Vitamin B_6_ Intake (Diet and Supplements) in 2002, Cumulative Mean^a^

Variable	Vitamin B_6_ Intake, mg/d				
	<2	2-4.9	5-14.9	15-34.9	≥35
No. (%) of population^b^	8416 (13.7)	33 660 (54.8)	8022 (13.1)	5965 (9.7)	5382 (8.8)
Age, mean, y	67.2	68.1	68.8	68.3	66.5
Height at baseline, mean, cm	164	164	164	164	164
Current BMI, mean	26.8	26.7	26.6	26.5	26.3
Physical activity, MET, mean, h/wk^c^	13.8	17.4	18.7	18.8	19.3
Current smoker, %	13.9	7.0	6.3	6.5	5.8
Dietary intake,mean					
Vitamin B_6_, mg/d^d^	1.7	3.1	8.4	23.3	69.1
Vitamin B_12_, μg/d^d^	7.4	12.7	22.4	31.6	48.2
Calcium, mg/d^d^	885	1167	1321	1345	1469
Vitamin D, μg/d^d^	5.0	9.9	12.5	12.2	13.7
Retinol, μg/d^d^	557	1104	1520	1596	2068
Protein, g/d^d^	67.7	73.0	73.9	73.0	73.3
Caffeine, mg/d	266	231	215	217	200
Alcohol, g/d	6.2	5.4	5.5	5.4	5.4
Multivitamin supplements, %	17.0	72.7	83.6	77.0	74.5
Vitamin B_6_ supplements, %	2.1	0.9	11.9	25.4	45.7
Vitamin B complex, %	0	0	11.9	27.8	36.9
Vitamin B_12_ supplements, %	2.3	3.2	9.0	14.6	24.7
Difficulty climbing stairs or walking 1 block, %	6.2	5.7	6.2	6.5	6.6
≥2 Falls last year	7.1	8.1	8.4	9.1	9.2
Self-rated general health status not excellent, %	10.1	10.7	10.8	11.2	11.1
Cancer, %	15.7	17.2	17.8	18.2	17.6
Diabetes, %	9.4	9.7	9.5	10.2	9.5
Cardiovascular disease, %	12.2	12.3	12.9	12.9	12.3
Osteoporosis, %	21.5	24.3	25.4	25.9	27.2
Medication use, %					
Current postmenopausal hormone therapy	29.0	35.6	39.5	37.3	37.6
Thiazide-like diuretic	13.2	15.2	15.4	15.4	14.0
Furosemide diuretic	3.2	3.9	3.9	3.9	4.8
Oral corticosteroids	2.3	2.5	2.6	2.6	2.8

Abbreviations: BMI, body mass index (calculated as weight in kilograms divided by height in meters squared); MET, metabolic equivalent.

^a^ Values are means and percentages and were standardized to the age distribution in 2002.

^b^ Number of women participating in the 2002 questionnaire cycle.

^c^ Metabolic equivalent hours per week from discretionary physical activity (eg, 12 MET hours per week is equivalent to 4 hours per week of walking or 1 hour per week of running).

^d^ Cumulative mean daily intake from foods and supplements adjusted for total energy intake.

**Table 2.** Age and Age-Adjusted Characteristics of 61 445 Women in the Nurses’ Health Study Across Categories of Total Vitamin B_12_ Intake (Diet and Supplements) in 2002, Cumulative Mean^a^

Variable	Vitamin B_12_ Intake, μg/d				
	<5	5-9.9	10-19.9	20-29.9	≥30
No. (%) of population^b^	4820 (7.8)	19 888 (32.4)	21 940 (35.7)	6144 (10.0)	8653 (14.1)
Age, mean, y	66.5	68.3	68.6	67.7	66.9
Height at baseline, mean, cm	164	164	164	164	164
Current BMI, mean	26.3	26.7	26.7	26.7	26.4
Physical activity, MET, h/wk^c^	16.1	16.4	17.6	18.0	19.1
Current smoker, %	9.8	8.3	7.8	6.9	5.8
Dietary intake, mean					
Vitamin B_6_, mg/d^d^	3.8	4.6	7.6	16.4	36.7
Vitamin B_12_, μg/d^d^	4.0	7.5	13.8	24.1	57.1
Calcium, mg/d^d^	886	1061	1244	1344	1423
Vitamin D, μg/d^d^	4.6	7.7	11.5	12.8	13.4
Retinol, μg/d^d^	393	816	1378	1631	1882
Protein, g/d^d^	66.2	71.5	73.8	74.0	73.2
Caffeine, mg/d	245	243	228	221	203
Alcohol, g/d	6.3	5.6	5.5	5.5	4.2
Multivitamin supplements, %	16.8	51.4	82.8	85.1	77.9
Vitamin B_6_ supplements, %	3.0	2.9	5.1	10.5	32.7
Vitamin B complex, %	0.1	0.2	3.7	17.5	31.2
Vitamin B_12_ supplements, %	0.1	0.1	0.2	3.1	47.4
Difficulty climbing stairs or walking 1 block, %	5.3	5.5	6.2	6.2	6.8
≥2 Falls last year	6.5	7.9	8.4	8.8	8.9
Self-rated general health status not excellent, %	9.2	10.6	10.9	11.2	11.3
Cancer, %	15.1	16.6	17.6	18.0	18.1
Diabetes, %	7.8	9.6	9.9	9.7	9.8
Cardiovascular disease, %	12.0	12.6	12.0	12.4	13.9
Osteoporosis, %	21.6	23.3	25.7	24.1	26.0
Medication use, %					
Current postmenopausal hormone therapy	30.9	33.6	36.9	35.6	38.6
Thiazide-like diuretic	12.9	14.4	15.6	15.3	14.7
Furosemide diuretic	3.3	3.6	4.1	3.9	4.5
Oral corticosteroids	2.5	2.2	2.6	2.8	2.7

Abbreviations: BMI, body mass index (calculated as weight in kilograms divided by height in meters squared); MET, metabolic equivalent.

^a^ Values are means and percentages and were standardized to the age distribution in 2002.

^b^ Number of women participating in the 2002 questionnaire cycle.

^c^ Metabolic equivalent hours per week from discretionary physical activity (eg, 12 MET hours per week is equivalent to 4 hours per week of walking or 1 hour per week of running).

^d^ Cumulative mean daily intake from foods and supplements adjusted for total energy intake.

The mean follow-up time was 20.9 years (1 586 155 person-years of follow-up). Compared with the reference category of total vitamin B_6 _less than 2 mg/d, an intake at least 35 mg/d was associated with an increased risk of hip fracture after adjusting for all covariates (RR, 1.29; 95% CI, 1.04-1.59; *P* = .06 for linear trend) (Table 3). For vitamin B_6_ from supplements only, those consuming no vitamin B_6_ supplements had the lowest risk compared with a similar increased risk in the other groups. For total vitamin B_12_, intakes at least 30 μg/d were associated with a nonsignificant increased risk of hip fracture compared with intakes less than 5 μg/d (RR, 1.25; 95% CI, 0.98-1.58), and risk increased linearly with increasing intake (RR, 1.01; 95% CI, 1.00-1.03 per 10-μg/d increase in total intake; *P* for linear trend = .02). Similar results were found for vitamin B_12_ intakes from supplements only (Table 3). The interaction term for the 2 vitamins on fracture risk was not significant. In analyses that included mutual adjustment for total B_6_ and B_12_ intakes, the associations were somewhat attenuated, with an RR of 1.19 (95% CI, 0.95-1.49) for vitamin B_6_ at least 35 mg/d vs less than 2 mg/d and an RR of 1.22 (95% CI, 0.94-1.57) for vitamin B_12_ at least 30 μg/d vs less than 5 μg/d. In fully adjusted models that included adjustment for intake from supplements, there was no clear association between vitamin B_6_ from diet only and hip fracture (RR, 1.03; 95% CI, 0.91-1.16 per 1-mg/d increase in intake from food only; *P* for linear trend = .67) or between vitamin B_12_ from diet only and hip fracture (RR, 1.01; 95% CI, 0.99-1.02 per 1-μg/d increase in intake from food only; *P* for linear trend = .54).

**Table 3.** Relative Risk of Hip Fracture According to Intake of Total Vitamins B_6_ and B_12_ and From Supplements Only Among Women With 2304 Hip Fractures, the Nurses’ Health Study, 1984-2014, Cumulative Mean

Variable	Cases	Crude Incidence per 10 000 Person-Years	RR (95% CI)	
			Age and Questionnaire Cycle Adjusted	Fully Adjusted^a^
Total vitamin B_6_, diet and supplements, mg/d				
<2	315	9.8	1 [Reference]	1 [Reference]
2-4.9	1242	15.1	1.06 (0.94-1.21)	1.11 (0.95-1.29)
5-14.9	353	17.4	1.13 (0.97-1.33)	1.17 (0.97-1.41)
15-34.9	200	17.3	1.07 (0.89-1.29)	1.10 (0.89-1.35)
≥35	194	16.1	1.22 (1.01-1.46)	1.29 (1.04-1.59)
*P* value for linear trend	NA	NA	.04	.06
RR per 10-mg/d increase	NA	NA	1.02 (1.00-1.04)	1.02 (1.00-1.04)
Vitamin B_6_, supplements only, mg/d				
0	123	5.2	1 [Reference]	1 [Reference]
<2	1177	16.2	1.37 (1.12-1.67)	1.37 (1.12-1.69)
2-4.9	424	14.4	1.37 (1.11-1.69)	1.31 (1.04-1.65)
5-24.9	353	19.7	1.50 (1.20-1.86)	1.44 (1.14-1.82)
≥25	227	15.1	1.42 (1.13-1.78)	1.41 (1.10-1.80)
*P* value for linear trend	NA	NA	.06	.09
RR per 10-mg/d increase	NA	NA	1.02 (1.00-1.04)	1.02 (1.00-1.04)
Total vitamin B_12_, diet and supplements, µg/d				
<5	162	9.8	1 [Reference]	1 [Reference]
5-9.9	687	12.1	1.00 (0.84-1.19)	1.01 (0.83-1.24)
10-19.9	888	15.9	1.12 (0.95-1.33)	1.09 (0.87-1.36)
20-29.9	230	17.3	1.21 (0.99-1.49)	1.21 (0.94-1.56)
≥30	337	21.4	1.27 (1.04-1.54)	1.25 (0.98-1.58)
*P* value for linear trend	NA	NA	.004	.02
RR per 10-μg/d increase	NA	NA	1.02 (1.01-1.03)	1.01 (1.00-1.03)
Vitamin B_12_, supplements only, μg/d				
0	161	8.1	1 [Reference]	1 [Reference]
<5	969	13.2	1.08 (0.91-1.28)	1.06 (0.89-1.26)
5-9.9	486	15.8	1.15 (0.96-1.38)	1.10 (0.89-1.35)
10-24.9	369	18.9	1.20 (0.99-1.46)	1.16 (0.94-1.44)
≥25	319	21.0	1.31 (1.07-1.59)	1.26 (1.01-1.56)
*P* value for linear trend	NA	NA	.03	.09
RR per 10-μg/d increase	NA	NA	1.01 (1.00-1.03)	1.01 (1.00-1.02)

Abbreviations: NA, not applicable; RR, relative risk.

^a^ Adjusted for age; questionnaire cycle; height; body mass index; physical activity; smoking status; dietary intakes of calcium, vitamin D, retinol, protein, caffeine, and alcohol; cancer; diabetes; cardiovascular disease; osteoporosis; postmenopausal hormone therapy; and use of thiazide diuretics, furosemide diuretics, and oral corticosteroids. In addition, vitamin B_12_ from supplements is adjusted for vitamin B_12_ from foods, and vitamin B_6_ from supplements is adjusted for vitamin B_6_ from foods.

Women with a high intake of both vitamins had a significantly increased risk of hip fracture compared with the reference category of a low intake of both vitamins (RR, 1.47; 95% CI, 1.15-1.89) (Table 4). Among women in the medium-intake categories for both vitamins, risk was not significantly elevated (RR, 1.18; 95% CI, 0.98-1.42). Few women had low intakes of one vitamin and high intakes of the other.

**Table 4.** Relative Risk of Hip Fracture According to Combined Cumulative Mean Total Intakes of Vitamins B_6_ and B_12_ Among Women With 2304 Hip Fractures, the Nurses’ Health Study, 1984-2014

Variable^a^	Cases	Crude Incidence per 10 000 Person-Years	Age and Questionnaire Cycle–Adjusted RR (95% CI)	Fully Adjusted RR (95% CI)^b^
Low B_6_ and low B_12_	263	9.5	1 [Reference]	1 [Reference]
Medium B_6_ and low B_12_	564	12.8	1.02 (0.88-1.19)	1.11 (0.94-1.31)
High B_6_ and low B_12_	22	11.2	1.19 (0.77-1.85)	1.27 (0.82-1.98)
Low B_6_ and medium B_12_	42	10.7	1.24 (0.89-1.74)	1.12 (0.79-1.59)
Medium B_6_ and medium B_12_	812	16.5	1.14 (0.99-1.32)	1.18 (0.98-1.42)
High B_6_ and medium B_12_	34	12.4	1.10 (0.77-1.58)	1.17 (0.80-1.72)
Low B_6_ and high B_12_	10	15.6	1.30 (0.69-2.45)	1.17 (0.62-2.22)
Medium B_6_ and high B_12_	419	19.8	1.25 (1.06-1.47)	1.31 (1.07-1.60)
High B_6_ and high B_12_	138	18.9	1.33 (1.08-1.65)	1.47 (1.15-1.89)

Abbreviation: RR, relative risk.

^a^ Cutoffs for vitamin B_6_ are 2 and 35 mg/d; cutoffs for vitamin B_12_ are 10 and 20 μg/d.

^b^ Adjusted for age; questionnaire cycle; height; body mass index; physical activity; smoking status; dietary intakes of calcium, vitamin D, retinol, protein, caffeine, and alcohol; cancer; diabetes; cardiovascular disease; osteoporosis; postmenopausal hormone therapy; and use of thiazide diuretics, furosemide diuretics, and oral corticosteroids.

There was a significant interaction between total vitamin B_6_ intake and BMI on hip fracture risk, while the interaction between total vitamin B_12 _intake and BMI was not significant. The results for total vitamins B_6_ and B_12_ intakes stratified by BMI are summarized in Table 5. The associations between vitamin B_6_ and vitamin B_12_ and hip fracture were stronger in women with BMI less than 25 compared with the overall analyses, whereas there were no clear associations in women with BMI at least 25. Among women with BMI less than 25, those with a high intake of both vitamins B_6_ and B_12_ had an RR of 1.71 (95% CI, 1.25-2.34) for hip fracture compared with those having a low intake of both vitamins. The corresponding RR in women with BMI at least 25 was 1.04 (95% CI, 0.66-1.64).

**Table 5.** Relative Risk of Hip Fracture According to Cumulative Mean Intake of B Vitamins Stratified by Body Mass Index Among Women With 2257 Hip Fractures, the Nurses’ Health Study, 1984-2014^a^

Variable	Cases	Fully Adjusted RR (95% CI)^b^
**BMI <25 (1409 Hip Fractures)**		
Total vitamin B_6_, mg/d		
<2	196	1 [Reference]
2-4.9	736	1.08 (0.89-1.31)
5-14.9	216	1.14 (0.90-1.44)
15-34.9	125	1.10 (0.84-1.43)
≥35	136	1.48 (1.13-1.93)
*P* value for linear trend	NA	.006
RR per 10-mg/d increase	NA	1.03 (1.01-1.06)
Total vitamin B_12_, μg/d		
<5	99	1 [Reference]
5-9.9	421	1.22 (0.94-1.58)
10-19.9	547	1.31 (0.99-1.74)
20-29.9	119	1.24 (0.89-1.72)
≥30	223	1.51 (1.11-2.04)
*P* value for linear trend	NA	.03
RR per 10-mg/d increase	NA	1.02 (1.00-1.03)
**BMI ≥25 (842 Hip Fractures)**		
Total vitamin B_6_, mg/d		
<2	112	1 [Reference]
2-4.9	476	1.10 (0.86-1.41)
5-14.9	130	1.19 (0.88-1.62)
15-34.9	69	1.06 (0.75-1.50)
≥35	55	1.01 (0.70-1.47)
*P* value for linear trend	NA	.93
RR per 10-mg/d increase	NA	1.00 (0.96-1.04)
Total vitamin B_12_, μg/d		
<5	60	1 [Reference]
5-9.9	253	0.76 (0.54-1.07)
10-19.9	321	0.78 (0.54-1.13)
20-29.9	104	1.08 (0.71-1.64)
≥30	104	0.81 (0.53-1.23)
*P* value for linear trend	NA	.62
RR per 10-mg/d increase	NA	1.01 (0.98-1.03)

Abbreviations: BMI, body mass index (calculated as weight in kilograms divided by height in meters squared); NA, not applicable; RR, relative risk.

^a^ Some individuals had missing BMI and were excluded from the analyses.

^b^ Adjusted for age; questionnaire cycle; height; BMI (as continuous variable); physical activity; smoking status; dietary intakes of calcium, vitamin D, retinol, protein, caffeine, and alcohol; cancer; diabetes; cardiovascular disease; osteoporosis; postmenopausal hormone therapy; and use of thiazide diuretics, furosemide diuretics, and oral corticosteroids.

We observed no significant interactions between total vitamin B_6_ or vitamin B_12_ intakes and age, indicating that the proportional hazards assumption was met. Similarly, there was no significant interaction between total vitamin B_6_ intake and physical activity for fracture risk. However, a significant interaction was found between total vitamin B_12_ intake and physical activity. There was a significant linear trend with increasing intake among those with a physical activity level below the median (RR, 1.02; 95% CI, 1.00-1.03 per 10-μg/d increase in total intake; *P* = .02) but not among those with a higher physical activity level (RR, 1.01; 95% CI, 0.99-1.03 per 10-μg/d increase in total intake; *P* = .51). Stratified results are summarized in eTable 1 in the Supplement.

In additional analyses of the data in Table 3, the following had little influence on the estimates: adjustment for BMI 4 years before the last follow-up and later weight change, difficulty climbing a flight of stairs or walking 1 block, difficulty with balance, falls in the past year, self-reported general health status, and pernicious anemia (eTable 2 in the Supplement). Adjustment for total folate intake had only a small influence on the associations between vitamin B_6_, vitamin B_12_, and hip fracture (eTable 3 in the Supplement).

In sensitivity analyses censoring participants at age 80 years, the association for total vitamin B_6_ intake was marginally stronger, whereas the results for total vitamin B_12_ intake were similar to the findings of the main analyses. The association for combined intakes of vitamins B_6_ and B_12_ as analyzed in Table 4 was also strengthened, with an RR of 1.74 (95% CI, 1.30-2.32) for women with a high intake of both vitamins.

In analyses conducted using current intakes of total vitamins B_6_ and B_12_, a similar pattern was seen. However, it was somewhat weaker compared with the analysis using cumulative average intakes (eTable 4 and eTable 5 in the Supplement).

## Discussion

We found that high intakes of vitamins B_6_ and B_12_ were associated with increased hip fracture risk among postmenopausal women in the Nurses’ Health Study. The risk was highest in women with a combined high intake of both vitamins, exhibiting an almost 50% increased risk of hip fracture compared with women with a low intake of both vitamins. High intakes were due to use of supplements. These findings add to previous studies suggesting that vitamin supplements should be used cautiously because adverse effects can occur.

### Other Studies

Our findings are in line with the results of a secondary analysis of combined data from 2 Norwegian double-blind RCTs with an identical intervention (6837 participants) in which an unexpected increased risk of hip fracture was found in those treated with high doses of vitamin B_6_ during an extended follow-up.^15^ Both RCTs had a 2 × 2 factorial design: the 4 groups were given (1) folic acid (0.8 mg) plus vitamin B_12_ (0.4 mg) and vitamin B_6_ (40 mg), (2) folic acid (0.8 mg) plus vitamin B_12_ (0.4 mg), (3) vitamin B_6_ alone (40 mg), or (4) placebo. Risk of hip fracture was highest in the first group (hazard ratio, 1.49; 95% CI, 1.05-2.11 compared with placebo).

We are not aware of other RCTs studying fractures that have supplemented with vitamin B_6_ alone, but several trials^14^ have combined vitamins B_6_ and B_12_. As summarized in eTable 6 in the Supplement, the results from the RCTs are divergent, as are the doses used and fracture type studied, making it difficult to draw firm conclusions. In the Women’s Antioxidant and Folic Acid Cardiovascular Study (WAFACS),^21^ there was no significant effect of the intervention (50 mg/d of vitamin B_6_ and 1 mg/d of vitamin B_12_ plus 2.5 mg/d of folic acid) on nonvertebral fractures (RR, 1.08; 95% CI, 0.88-1.34). A statistical interaction with baseline plasma B_6_ concentration was observed; when excluding women in the lower quartile of baseline vitamin B_6_, there was a significantly increased risk of nonvertebral fractures (RR, 1.39; 95% CI, 1.01-1.91). The Heart Outcomes Prevention Evaluation (HOPE) 2 trial^22^ intervened with 50 mg/d of vitamin B_6_, 1 mg/d of vitamin B_12_, and 2.5 mg/d of folic acid. There was a nonsignificant hazard ratio of 1.07 (95% CI, 0.85-1.33) for nonvertebral fractures. The Vitamins to Prevent Stroke (VITATOPS) trial^23^ gave a lower dose of vitamin B_6_ (25 mg/d) in addition to 0.5 mg/d of vitamin B_12_ plus 2.0 mg/d of folic acid. The authors reported no significant effect on any osteoporotic fracture (n = 145) (hazard ratio, 0.86; 95% CI, 0.62-1.18) or hip fracture (n = 70) (hazard ratio, 0.94; 95% CI, 0.59-1.50).

### Possible Mechanisms

A possible biological explanation for the findings in the present study is not clear. The magnitude of intakes of vitamins B_6_ andB_12_ associated with an increased risk of hip fracture in our study far exceeded the recommended dietary allowances (RDAs) (1.3-1.7 mg/d for vitamin B_6_ and 2.4 μg/d for vitamin B_12_).^15^

Possible adverse effects of high-dose vitamin B_6_ supplementation have previously been suggested.^24,25^ High doses (≥500 mg/d) might increase the risk of falling because neurological symptoms, including ataxia, neuropathy, and decreased muscle tone, have been reported, and milder neurological symptoms have been observed at doses of approximately 100 mg/d as adverse effects.^26^ Preliminary work suggested that high vitamin B_6_ concentrations might accelerate bone loss by counteracting the modulating influence of estrogens on steroid receptors.^27^ A recent paradox theory proposes that large doses of pyridoxine, the inactive form of vitamin B_6_ included in supplements and found in foods, inhibits the active form pyridoxal phosphate.^28^

We do not have an explanation for the mechanism by which vitamin B_12_ may contribute to increased fracture risk. However, as summarized in Table 4 and in eTable 5 in the Supplement, a high intake of vitamin B_12_ and a low intake of vitamin B_6_ were not associated with increased risk, which is in agreement with a meta-analysis^14^ of RCTs giving vitamin B_12_ and/or folic acid alone (without vitamin B_6_).

A possible explanation for the interaction between total vitamin B_6_ intake and BMI is not clear. At low BMI, fracture risk was higher, and a larger number of incident hip fractures thus occurred, yielding higher statistical power. We speculate that the possible mechanism of excessive vitamin B_6_ exposure increasing fall risk through neurological symptoms could particularly aggravate fracture risk in women with low BMI, who are more prone to fracturing their hip when experiencing a fall. However, adjustment for falls had little influence on the estimates, and the association between falls and fracture risk was not altered by adjustment for vitamins B_6_ and B_12_ intakes. It also could be that the possible interaction between vitamin B_6_ and the steroid receptor might be most influential in lean women, who have a reduced capacity for production of adipose-derived estrogens.^29^

### Strengths and Limitations

Our study has strengths and limitations. We were able to follow up a large cohort of women with repeated detailed assessments of diet, supplement use, and other possible confounding factors. However, we cannot exclude the possibility that individuals started taking supplements due to ill health. In addition, all information on vitamin intake and confounders was collected by questionnaires, with their inherent limitations. Although residual confounding could be present, our results were not substantially influenced by adjustment for indicators of frailty or disease or a long list of other possible confounders. Another limitation was the self-report of hip fractures. However, in sensitivity analyses censoring participants at age 80 years (thus omitting the oldest, in whom underreporting of fracture could be an issue), the association for total vitamin B_6_ intake was marginally stronger. An additional limitation is that the findings may be applicable only to women of white race/ethnicity.

The results for supplemental vitamin B_6_ are puzzling because all categories above the reference category have similar increases in risk. Also, intakes of different supplements are correlated, making it challenging to disentangle specific associations. Nevertheless, our results were adjusted for intake of calcium, vitamin D, and retinol, and the differences between the model adjusted for age and questionnaire cycle and the fully adjusted models were modest.

We did not control for multiple hypothesis testing. However, our analyses were based on the RCT results, in which the highest fracture risk was found in those treated with high doses of both vitamins.^15^ A low proportion of the women were in the low-intake category of both vitamins. Yet, compared with the group with a medium intake of both vitamin B_6_ (2 to <35 mg/d) and vitamin B_12_ (10 to <20 μg/d), the risk was still significantly increased in those with a high intake of both vitamins (RR, 1.25; 95% CI, 1.03-1.51).

### Implications

The RDAs are established to meet the nutritional requirements of almost the entire population. Despite that, use of high-dose vitamin supplementation far exceeding the RDAs is common, often without any definite indication and in the absence of clear evidence of benefit.

Our results are in line with several reports suggesting that unexpected adverse effects can occur with high-dose vitamin supplementation. For example, high-dose beta-carotene supplementation increased the risk of lung cancer in smokers,^3^ and high-dose vitamin E supplementation may increase all-cause mortality.^4^ Higher risk of fracture was reported in 2 RCTs^5,6^ after treatment with annual megadoses of vitamin D, and possible adverse effects of homocysteine-lowering treatment with B vitamins have been observed,^9,11^ including a potentially increased risk of cancer.^12^ Although we acknowledge the limitations of our cohort design, the findings herein add to the body of literature that suggests caution should be used in vitamin supplementation when there is no apparent deficiency.

## Conclusions

In this large prospective cohort study of postmenopausal women in the Nurses’ Health Study, we found that a combined high intake of vitamins B_6_ and B_12_ was associated with an increased risk of hip fracture. These findings add to previous studies suggesting that vitamin supplements should be used cautiously because adverse effects can occur.

## Notes

Supplement. **eFigure.** Flow of Inclusion for the Analyses of Vitamins B_6_ and B_12_ and Hip Fracture in the Nurse’s Health Study Starting in 1984 **eTable 1.** Relative Risk (RR) of Hip Fracture According to Intake of Vitamin B_12_ Among Women, the Nurses’ Health Study (1986-2014) **eTable 2.** Relative Risk (RR) of Hip Fracture According to Cumulative Average Intake of Vitamins B_6_ and B_12_ Among Women, the Nurses’ Health Study **eTable 3.** Relative Risk (RR) of Hip Fracture According to Cumulative Average Intake of Vitamin B_6_, Vitamin B_12_, and Folate Among Women, the Nurses’ Health Study (1986-2014) (2,304 Hip Fractures) **eTable 4.** Relative Risk (RR) of Hip Fracture According to Intakes of Vitamins B_6_ and B_12_ Among Women, the Nurses’ Health Study (1986-2014) (2,812 Hip Fractures), Current Intake **eTable 5.** Relative Risk (RR) of Hip Fracture According to Current Intake of Vitamins B_6_ and B_12_ Among Women, the Nurses’ Health Study (1986-2014) (2,812 Hip Fractures) **eTable 6.** Randomized Controlled Trials Intervening With Both Vitamin B_6_ and B_12_ and Reporting on the Risk of Fracture **eReferences.**

## References

[1] Kantor ED, Rehm CD, Du M, White E, Giovannucci EL Trends in dietary supplement use among US adults from 1999-2012. JAMA. 2016;316(14):-. 27727382 10.1001/jama.2016.14403 PMC5540241

[2] Kim HJ, Giovannucci E, Rosner B, Willett WC, Cho E Longitudinal and secular trends in dietary supplement use: Nurses’ Health Study and Health Professionals Follow-Up Study, 1986-2006. J Acad Nutr Diet. 2014;114(3):436-443. 24119503 10.1016/j.jand.2013.07.039 PMC3944223

[3] Druesne-Pecollo N, Latino-Martel P, Norat T, Beta-carotene supplementation and cancer risk: a systematic review and metaanalysis of randomized controlled trials. Int J Cancer. 2010;127(1):172-184. 19876916 10.1002/ijc.25008

[4] Miller ER III, Pastor-Barriuso R, Dalal D, Riemersma RA, Appel LJ, Guallar E Meta-analysis: high-dosage vitamin E supplementation may increase all-cause mortality. Ann Intern Med. 2005;142(1):37-46. 15537682 10.7326/0003-4819-142-1-200501040-00110

[5] Sanders KM, Stuart AL, Williamson EJ, Annual high-dose oral vitamin D and falls and fractures in older women: a randomized controlled trial. JAMA. 2010;303(18):1815-1822. 20460620 10.1001/jama.2010.594

[6] Smith H, Anderson F, Raphael H, Maslin P, Crozier S, Cooper C Effect of annual intramuscular vitamin D on fracture risk in elderly men and women: a population-based, randomized, double-blind, placebo-controlled trial. Rheumatology (Oxford). 2007;46(12):1852-1857. 17998225 10.1093/rheumatology/kem240

[7] Zhang C, Wang ZY, Qin YY, Yu FF, Zhou YH Association between B vitamins supplementation and risk of cardiovascular outcomes: a cumulative meta-analysis of randomized controlled trials. PLoS One. 2014;9(9):e107060. 25238614 10.1371/journal.pone.0107060 PMC4169527

[8] Myung SK, Ju W, Cho B, ; Korean Meta-Analysis Study Group Efficacy of vitamin and antioxidant supplements in prevention of cardiovascular disease: systematic review and meta-analysis of randomised controlled trials. BMJ. 2013;346:f10. 23335472 10.1136/bmj.f10 PMC3548618

[9] Clarke R, Halsey J, Lewington S, ; B-Vitamin Treatment Trialists’ Collaboration Effects of lowering homocysteine levels with B vitamins on cardiovascular disease, cancer, and cause-specific mortality: meta-analysis of 8 randomized trials involving 37 485 individuals. Arch Intern Med. 2010;170(18):1622-1631. 20937919 10.1001/archinternmed.2010.348

[10] Vollset SE, Clarke R, Lewington S, ; B-Vitamin Treatment Trialists’ Collaboration Effects of folic acid supplementation on overall and site-specific cancer incidence during the randomised trials: meta-analyses of data on 50,000 individuals. Lancet. 2013;381(9871):1029-1036. 23352552 10.1016/S0140-6736(12)62001-7 PMC3836669

[11] Løland KH, Bleie O, Blix AJ, Effect of homocysteine-lowering B vitamin treatment on angiographic progression of coronary artery disease: a Western Norway B Vitamin Intervention Trial (WENBIT) substudy. Am J Cardiol. 2010;105(11):1577-1584. 20494665 10.1016/j.amjcard.2010.01.019

[12] van Wijngaarden JP, Swart KM, Enneman AW, Effect of daily vitamin B-12 and folic acid supplementation on fracture incidence in elderly individuals with an elevated plasma homocysteine concentration: B-PROOF, a randomized controlled trial. Am J Clin Nutr. 2014;100(6):1578-1586. 25411293 10.3945/ajcn.114.090043

[13] Herrmann M, Peter Schmidt J, Umanskaya N, The role of hyperhomocysteinemia as well as folate, vitamin B_6_ and B_12_ deficiencies in osteoporosis: a systematic review. Clin Chem Lab Med. 2007;45(12):1621-1632. 18067447 10.1515/CCLM.2007.362

[14] Garcia Lopez M, Baron JA, Omsland TK, Søgaard AJ, Meyer HE Homocysteine-lowering treatment and the risk of fracture: secondary analysis of a randomized controlled trial and an updated meta-analysis. JBMR Plus. 2018;2(5):295-303. 30283911 10.1002/jbm4.10045 PMC6139704

[15] Garcia Lopez M, Bønaa KH, Ebbing M, B vitamins and hip fracture: secondary analyses and extended follow-up of two large randomized controlled trials. J Bone Miner Res. 2017;32(10):1981-1989. 28574605 10.1002/jbmr.3189

[16] Colditz GA, Martin P, Stampfer MJ, Validation of questionnaire information on risk factors and disease outcomes in a prospective cohort study of women. Am J Epidemiol. 1986;123(5):894-900. 3962971 10.1093/oxfordjournals.aje.a114319

[17] Willett WC *Nutritional Epidemiology* 3rd ed. New York, NY: Oxford University Press; 2013.

[18] Yuan C, Spiegelman D, Rimm EB, Validity of a dietary questionnaire assessed by comparison with multiple weighed dietary records or 24-hour recalls. Am J Epidemiol. 2017;185(7):570-584. 28338828 10.1093/aje/kww104 PMC5859994

[19] Zhang SM, Willett WC, Selhub J, Plasma folate, vitamin B_6_, vitamin B_12_, homocysteine, and risk of breast cancer. J Natl Cancer Inst. 2003;95(5):373-380. 12618502 10.1093/jnci/95.5.373

[20] Ainsworth BE, Haskell WL, Leon AS, Compendium of physical activities: classification of energy costs of human physical activities. Med Sci Sports Exerc. 1993;25(1):71-80. 8292105 10.1249/00005768-199301000-00011

[21] Stone KL, Lui LY, Christen WG, Effect of combination folic acid, vitamin B_6_, and vitamin B_12_ supplementation on fracture risk in women: a randomized, controlled trial. J Bone Miner Res. 2017;32(12):2331-2338. 29244251 10.1002/jbmr.3229 PMC5734110

[22] Sawka AM, Ray JG, Yi Q, Josse RG, Lonn E Randomized clinical trial of homocysteine level–lowering therapy and fractures. Arch Intern Med. 2007;167(19):2136-2139. 17954810 10.1001/archinte.167.19.2136

[23] Gommans J, Yi Q, Eikelboom JW, Hankey GJ, Chen C, Rodgers H; VITATOPS Trial Study Group The effect of homocysteine-lowering with B-vitamins on osteoporotic fractures in patients with cerebrovascular disease: substudy of VITATOPS, a randomised placebo-controlled trial. BMC Geriatr. 2013;13:88. 24004645 10.1186/1471-2318-13-88 PMC3848681

[24] Collier J Vitamin B-6: food or medicine? the rules—and the politics—are different. BMJ. 1998;317(7151):92-93. 9657780 10.1136/bmj.317.7151.92 PMC1113521

[25] Still time for rational debate about vitamin B_6_. Lancet. 1998;351(9115):1523. 10326528

[26] Dalton K, Dalton MJ Characteristics of pyridoxine overdose neuropathy syndrome. Acta Neurol Scand. 1987;76(1):8-11. 3630649 10.1111/j.1600-0404.1987.tb03536.x

[27] Allgood VE, Cidlowski JA Vitamin B_6_ modulates transcriptional activation by multiple members of the steroid hormone receptor superfamily. J Biol Chem. 1992;267(6):3819-3824. 1310983

[28] Vrolijk MF, Opperhuizen A, Jansen EHJM, Hageman GJ, Bast A, Haenen GRMM The vitamin B_6_ paradox: supplementation with high concentrations of pyridoxine leads to decreased vitamin B_6_ function. Toxicol In Vitro. 2017;44:206-212. 28716455 10.1016/j.tiv.2017.07.009

[29] Shapses SA, Sukumar D Bone metabolism in obesity and weight loss. Annu Rev Nutr. 2012;32:287-309. 22809104 10.1146/annurev.nutr.012809.104655 PMC4016236

