# Supplementary material for: Association of High Intakes of Vitamins B6 and B12 From Food and Supplements With Risk of Hip Fracture Among Postmenopausal Women in the Nurses’ Health Study
Source: JAMA Netw Open. 2019 May 10;2(5):e193591. doi: 10.1001/jamanetworkopen.2019.3591 (PMC6512300; doi:10.1001/jamanetworkopen.2019.3591)
Supplement: Supplement. — eFigure. Flow of Inclusion for the Analyses of Vitamins B6 and B12 and Hip Fracture in the Nurse’s Health Study Starting in 1984 eTable 1. Relative Risk (RR) of Hip Fracture According to Intake of Vitamin B12 Among Women, the Nurses’ Health Study (1986-2014) eTable 2. Relative Risk (RR) of Hip Fracture According to Cumulative Average Intake of Vitamins B6 and B12 Among Women, the Nurses’ Health Study eTable 3. Relative Risk (RR) of Hip Fracture According to Cumulative Average Intake of Vitamin B6, Vitamin B12, and Folate Among Women, the Nurses’ Health Study (1986-2014) (2,304 Hip Fractures) eTable 4. Relative Risk (RR) of Hip Fracture According to Intakes of Vitamins B6 and B12 Among Women, the Nurses’ Health Study (1986-2014) (2,812 Hip Fractures), Current Intake eTable 5. Relative Risk (RR) of Hip Fracture According to Current Intake of Vitamins B6 and B12 Among Women, the Nurses’ Health Study (1986-2014) (2,812 Hip Fractures) eTable 6. Randomized Controlled Trials Intervening With Both Vitamin B6 and B12 and Reporting on the Risk of Fracture eReferences. [file jamanetwopen-2-e193591-s001.pdf]

Supplementary Online Content

Meyer HE, Willett WC, Fung TT, Holvik K, Feskanich D. Association of high intakes of vitamins B_6 and B_{12} from food and supplements with risk of hip fracture among postmenopausal women in the Nurses' Health Study *JAMA Netw Open.* 2019;2(5):e193591. doi:10.1001/jamanetworkopen.2019.3591

eFigure. Flow of Inclusion for the Analyses of Vitamins B_6 and B_{12} and Hip Fracture in the Nurse's Health Study Starting in 1984

eTable 1. Relative Risk (RR) of Hip Fracture According to Intake of Vitamin B_{12} Among Women, the Nurses' Health Study (1986-2014)

eTable 2. Relative Risk (RR) of Hip Fracture According to Cumulative Average Intake of Vitamins B_6 and B_{12} Among Women, the Nurses' Health Study

eTable 3. Relative Risk (RR) of Hip Fracture According to Cumulative Average Intake of Vitamin B_6, Vitamin B_{12}, and Folate Among Women, the Nurses' Health Study (1986-2014) (2,304 Hip Fractures)

eTable 4. Relative Risk (RR) of Hip Fracture According to Intakes of Vitamins B_6 and B_{12} Among Women, the Nurses' Health Study (1986-2014) (2,812 Hip Fractures), Current Intake

eTable 5. Relative Risk (RR) of Hip Fracture According to Current Intake of Vitamins B_6 and B_{12} Among Women, the Nurses' Health Study (1986-2014) (2,812 Hip Fractures)

eTable 6. Randomized Controlled Trials Intervening With Both Vitamins B_6 and B_{12} and Reporting on the Risk of Fracture

eReferences.

This supplementary material has been provided by the authors to give readers additional information about their work.

© 2019 Meyer et al. *JAMA Network Open.*

eFigure. **Flow of inclusion for the analyses of vitamins B_6 and B_{12} and hip fracture in the Nurse's Health Study, starting in 1984**

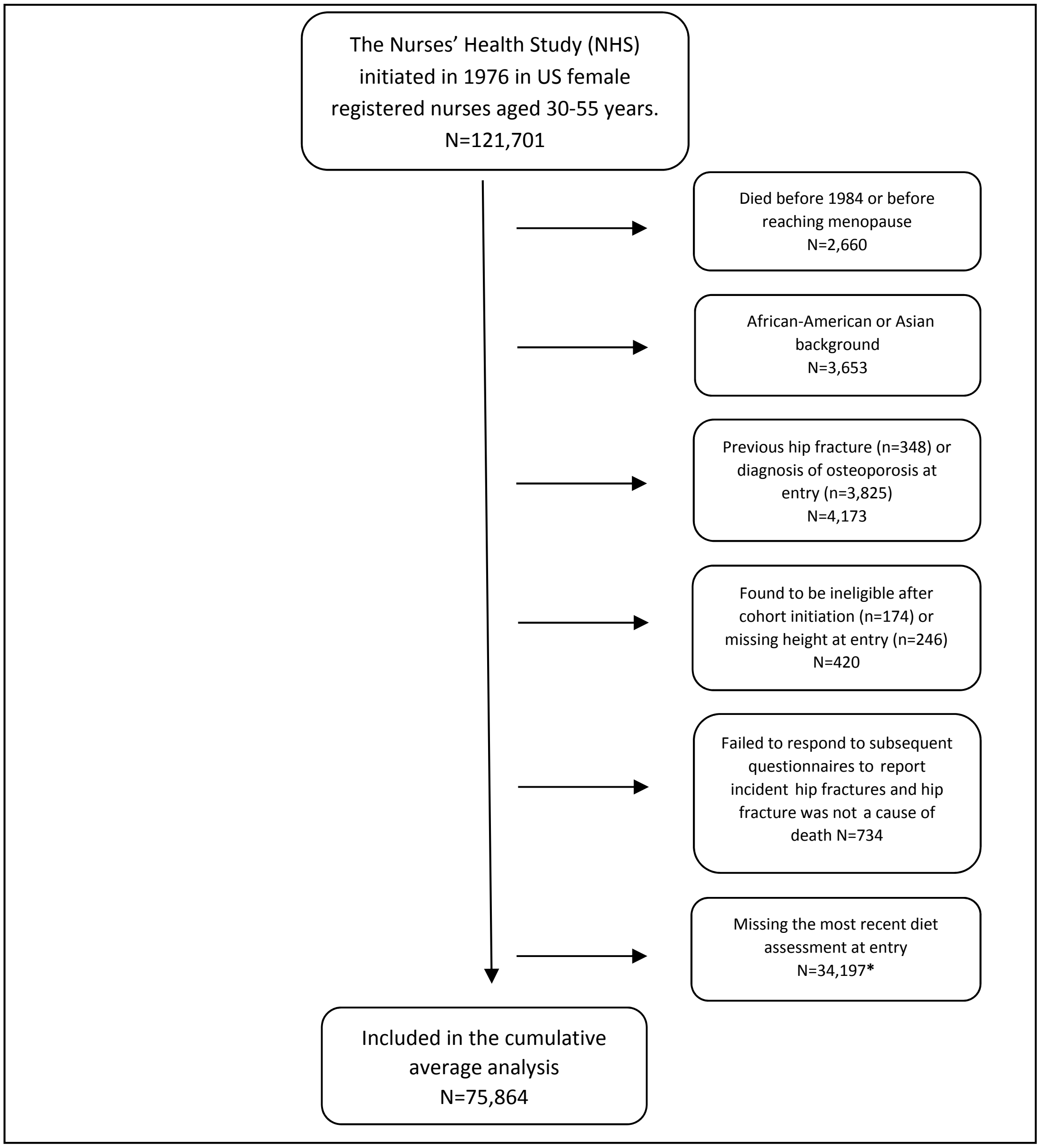

* As dietary information at entry was a requirement for constructing cumulative average intakes, the sample size was lower for the **analysis using cumulative average intakes (n=75,864, with 2,304 hip fracture)** than for the **analysis using current intakes (n=96,467, with 2,812 hip fractures)** where this was not a prerequisite.

© 2019 Meyer et al. *JAMA Network Open.*

eTable 1. Relative risk (RR) of hip fracture according to intake of vitamin B_{12} among women, the Nurses' Health Study (1984-2014). Cumulative average, stratified on physical activity.

Intake categories	Cases	RR (95% CI) (fully adjusted[a])
Low physical activity (< median) (1528 hip fractures)		
**Vitamin B_{12} total (µg/day)**		
< 5	119	1.00 (Ref)
5 –	478	0.88 (0.69-1.12)
10 –	579	0.93 (0.72-1.21)
20 –	157	1.14 (0.84-1.55)
≥ 30	195	1.06 (0.79-1.42)
P value for linear trend		*0.021*
High physical activity (≥ median) (723 hip fractures)		
**Vitamin B_{12} total (µg/day)**		
< 5	40	1.00 (Ref)
5 –	196	1.34 (0.90-2.01)
10 –	289	1.45 (0.94-2.23)
20 –	66	1.33 (0.81-2.18)
≥ 30	132	1.65 (1.04-2.61)
P value for linear trend		*0.51*

[a] Adjusted for age, questionnaire cycle, body mass index, height, physical activity, postmenopausal hormones, smoking status, cancer, cardiovascular disease, diabetes, osteoporosis, use of furosemide diuretics, thiazide diuretics, and oral steroids, and intakes of calcium, vitamin D, retinol, protein, caffeine, and alcohol.

© 2019 Meyer et al. *JAMA Network Open.*

eTable 2. Relative risk (RR) of hip fracture according to cumulative average intake of vitamins B_6 and B_{12} among women, the Nurses' Health Study. Additional analyses adjusting for body mass index (BMI) four years previously and later weight change, self-reported health status, difficulties climbing a flight of stairs or walking one block, difficulty with balance, falls in the past year or pernicious anemia.

Intake categories	RR (95% CI) ('fully' adjusted[a])	RR (95% CI) (additionally adjusted)
**1988-2014 (2090 hip fractures)**		
**Vitamin B_6 total (mg/day)**		*additionally adjusted for BMI 4 years ago and weight change* [b]
< 2	1.00	1.00
2 –	1.11	1.12 (0.95-1.31)
5 –	1.18	1.19 (0.98-1.44)
15 –	1.12	1.12 (0.90-1.39)
≥ 35	1.32	1.31 (1.05-1.64)
P value for linear trend	*0.021*	*0.025*
**Vitamin B_{12} total (µg/day)**		*additionally adjusted for BMI 4 years ago and weight change* [b]
< 5	1.00	1.00
5 –	1.04	1.04 (0.83-1.29)
10 –	1.11	1.12 (0.88-1.41)
20 –	1.26	1.25 (0.96-1.64)
≥ 30	1.27	1.27 (0.99-1.64)
P value for linear trend	*0.039*	*0.04*
**1992-2014 (2068 hip fractures)**		
**Vitamin B_6 total (mg/day)**		*additionally adjusted for health status* [c]
< 2	1.00	1.00
2 –	1.11	1.10 (0.94-1.30)
5 –	1.19	1.17 (0.96-1.43)
15 –	1.11	1.10 (0.89-1.37)
≥ 35	1.34	1.33 (1.06-1.67)
P value for linear trend	*0.013*	*0.013*
**Vitamin B_{12} total (µg/day)**		*additionally adjusted for health status* [c]
< 5	1.00	1.00
5 –	1.04	1.03 (0.82-1.28)
10 –	1.12	1.11 (0.87-1.41)
20 –	1.26	1.25 (0.95-1.64)
≥ 30	1.29	1.27 (0.98-1.64)
P value for linear trend	*0.013*	*0.019*
**1992-2014 (2068 hip fractures)**		
**Vitamin B_6 total (mg/day)**		*add. adj. for diff. climbing stairs, walking one block and balance* [d]
< 2	1.00	1.00
2 –	1.11	1.11 (0.94-1.31)
5 –	1.19	1.18 (0.97-1.44)
15 –	1.11	1.10 (0.88-1.36)
≥ 35	1.34	1.33 (1.06-1.66)
P value for linear trend	*0.013*	*0.018*

© 2019 Meyer et al. *JAMA Network Open.*

**Vitamin B_{12} total (µg/day)**		*add. adj. for diff. climbing stairs, walking one block and balance* [d]
< 5	1.00	1.00
5 –	1.04	1.02 (0.81-1.27)
10 –	1.12	1.10 (0.86-1.40)
20 –	1.26	1.24 (0.94-1.62)
≥ 30	1.29	1.24 (0.96-1.60)
P value for linear trend	*0.013*	*0.03*
**1998-2014 (1692 hip fractures)**		
**Vitamin B_6 total (mg/day)**		*additionally adjusted for falls* [e]
< 2	1.00	1.00
2 –	0.98	0.98 (0.81-1.19)
5 –	1.03	1.03 (0.82-1.30)
15 –	0.99	0.98 (0.77-1.26)
≥ 35	1.19	1.18 (0.92-1.53)
P value for linear trend	*0.035*	*0.039*
**Vitamin B_{12} total (µg/day)**		*additionally adjusted for falls* [e]
< 5	1.00	1.00
5 –	1.16	1.15 (0.87-1.52)
10 –	1.25	1.23 (0.92-1.65)
20 –	1.40	1.39 (1.01-1.91)
≥ 30	1.45	1.42 (1.05-1.93)
P value for linear trend	*0.009*	*0.015*
**1998-2014 (1692 hip fractures)**		
**Vitamin B_6 total (mg/day)**		*additionally adjusted for pernicious anemia* [f]
< 2	1.00	1.00
2 –	0.98	0.98 (0.81-1.19)
5 –	1.03	1.03 (0.82-1.30)
15 –	0.99	0.99 (0.78-1.26)
≥ 35	1.19	1.19 (0.92-1.53)
P value for linear trend	*0.035*	*0.035*
**Vitamin B_{12} total (µg/day)**		*additionally adjusted for pernicious anemia* [f]
< 5	1.00	1.00
5 –	1.16	1.16 (0.88-1.53)
10 –	1.25	1.24 (0.93-1.67)
20 –	1.40	1.39 (1.01-1.92)
≥ 30	1.45	1.43 (1.05-1.94)
P value for linear trend	*0.009*	*0.020*

[a] Adjusted for age, questionnaire cycle, BMI, height, physical activity, postmenopausal hormones, smoking status, cancer, cardiovascular disease, diabetes, osteoporosis, use of furosemide diuretics, thiazide diuretics, and oral steroids, and intakes of calcium, vitamin D, retinol, protein, caffeine, and alcohol

[b] Control for BMI 4 yrs ago and weight change over the past 4 yrs.

[c] Control for self-reported health status (agreement with "my health is excellent") - beginning in 1992

[d] Control for difficulty climbing a flight of stairs or walking one block and difficulty with balance - beginning in 1992

[e] Control for number of falls in the past year, beginning in 1998

[f] Control for pernicious anemia, beginning in 1998

© 2019 Meyer et al. *JAMA Network Open.*

eTable 3. Relative risk (RR) of hip fracture according to cumulative average intake of vitamin B_6, vitamin B_{12} and folate among women, the Nurses' Health Study (1984-2014) (2,304 hip fractures).

Intake categories	RR (95% CI) (fully adjusted[a])	RR (95% CI) (additionally adjusted[b])
**Folate, total (µg/day)**		*adjusted for vitamin B_6*
< 300	1.00	1.00
300 –	1.02	0.97 (0.82-1.15)
450 –	1.02	0.95 (0.78-1.16)
600 –	1.12	1.05 (0.84-1.30)
≥ 800	1.18	1.08 (0.84-1.40)
P value for linear trend	*0.052*	*0.12*
**Vitamin B_6 total (mg/day)**		*adjusted for folate*
< 2	1.00	1.00
2 –	1.11	1.13 (0.95-1.33)
5 –	1.17	1.18 (0.97-1.43)
15 –	1.10	1.11 (0.89-1.38)
≥ 35	1.29	1.29 (1.03-1.61)
P value for linear trend	*0.059*	*0.14*
**Folate, total (µg/day)**		*adjusted for vitamin B_{12}*
< 300	1.00	1.00
300 –	1.02	1.01 (0.87-1.18)
450 –	1.02	1.00 (0.83-1.20)
600 –	1.12	1.08 (0.88-1.32)
≥ 800	1.18	1.11 (0.87-1.42)
P value for linear trend	*0.052*	*0.13*
**Vitamin B_{12} total (µg/day)**		*adjusted for folate*
< 5	1.00	1.00
5 –	1.01	1.02 (0.83-1.24)
10 –	1.09	1.08 (0.87-1.35)
20 –	1.21	1.20 (0.93-1.55)
≥ 30	1.25	1.22 (0.96-1.56)
P value for linear trend	*0.017*	*0.044*

[a] Adjusted for age, questionnaire cycle, body mass index, height, physical activity, postmenopausal hormones, smoking status, cancer, cardiovascular disease, diabetes, osteoporosis, use of furosemide diuretics, thiazide diuretics, and oral steroids, and intakes of calcium, vitamin D, retinol, protein, caffeine, and alcohol

[b] Folate additionally adjusted for vitamin B12 or vitamin B6. Vitamin B12 and B6 additionally adjusted for folate

© 2019 Meyer et al. *JAMA Network Open.*

eTable 4. Relative risk (RR) of hip fracture according to intake of vitamins B_6 and B_{12} among women, the Nurses' Health Study (1984-2014) (2,812 hip fractures), current intake.

Intake categories	Cases	Crude incidence/10,000[a]	RR (95% CI) (age and cycle adjusted)	RR (95% CI) (fully adjusted[b])
**Vitamin B_6 total (mg/day)**				
< 2	490	12.1	1.00	1.00
2 –	1426	14.6	0.95 (0.86-1.06)	1.02 (0.90-1.15)
5 –	449	18.4	1.04 (0.91-1.19)	1.10 (0.94-1.29)
15 –	143	15.0	0.98 (0.81-1.18)	1.03 (0.84-1.27)
≥ 35	304	17.8	1.06 (0.92-1.23)	1.12 (0.95-1.33)
P value for linear trend			*0.09*	*0.11*
**Vitamin B_6 suppl. (mg/day)**				
0	733	11.4	1.00	1.00
< 2	589	14.8	1.14 (1.02-1.27)	1.23 (1.08-1.40)
2 –	927	18.1	1.14 (1.03-1.26)	1.24 (1.08-1.43)
5 –	150	15.1	1.27 (1.07-1.52)	1.37 (1.12-1.67)
≥ 25	413	17.3	1.19 (1.05-1.35)	1.29 (1.11-1.49)
P value for linear trend			*0.17*	*0.18*
**Vitamin B_{12} total (μg/day)**				
< 5	314	12.8	1.00	1.00
5 –	737	11.8	0.95 (0.83-1.09)	1.04 (0.89-1.22)
10 –	803	14.4	1.05 (0.92-1.19)	1.13 (0.94-1.34)
20 –	312	16.9	1.00 (0.85-1.18)	1.10 (0.91-1.35)
≥ 30	646	22.7	1.15 (1.00-1.33)	1.26 (1.05-1.51)
P value for linear trend			*<0.001*	*0.002*
**Vitamin B_{12} suppl. (μg/day)**				
0	502	13.6	1.00	1.00
<5	752	11.2	1.11 (0.99-1.25)	1.14 (1.00-1.29)
5 –	501	15.3	1.10 (0.97-1.24)	1.09 (0.93-1.28)
10 –	359	17.9	1.06 (0.93-1.22)	1.12 (0.96-1.32)
≥ 25	698	21.6	1.16 (1.03-1.31)	1.20 (1.04-1.39)
P value for linear trend			*0.002*	*0.006*

[a] Per 10 000 person-years

[b] Adjusted for age, questionnaire cycle, body mass index, height, physical activity, postmenopausal hormones, smoking status, cancer, cardiovascular disease, diabetes, osteoporosis, use of furosemide diuretics, thiazide diuretics, and oral steroids, and intakes of calcium, vitamin D, retinol, protein, caffeine, and alcohol

© 2019 Meyer et al. *JAMA Network Open.*

eTable 5. Relative risk (RR) of hip fracture according to combined current intakes of vitamins B_6 and B_{12} among women, the Nurses' Health Study (1984-2014) (2,812 hip fractures)

Categories of total vitamin B_6 and B_{12} intakes[a]	Cases	Person-years	RR (age and cycle adjusted)	RR (95% CI) (fully adjusted[b])
Low B_6 – Low B_{12}	425	352622	1.00	1.00 (ref)
Med B_6 – Low B_{12}	601	492799	0.91	0.99 (0.86-1.14)
High B_6 – Low B_{12}	25	23103	0.90	0.97 (0.64-1.46)
Low B_6 – Med B_{12}	43	42242	1.16	1.07 (0.76-1.49)
Med B_6 – Med B_{12}	736	489055	1.02	1.09 (0.93-1.27)
High B_6 – Med B_{12}	24	12532	0.83	0.88 (0.57-1.35)
Low B_6 – High B_{12}	22	5277	1.09	0.99 (0.64-1.53)
Med B_6 – High B_{12}	681	167870	1.04	1.13 (0.96-1.33)
High B_6 – High B_{12}	255	122185	1.15	1.23 (1.02-1.49)

[a] Cut-offs for vitamin B6: 2 and 35 mg/day; Cut-offs for vitamin B12: 10 and 20 µg/day

[b] Adjusted for age, questionnaire cycle, body mass index, height, physical activity, postmenopausal hormones, smoking status, cancer, cardiovascular disease, diabetes, osteoporosis, use of furosemide diuretics, thiazide diuretics, and oral steroids, and intakes of calcium, vitamin D, retinol, protein, caffeine, and alcohol

© 2019 Meyer et al. *JAMA Network Open.*

eTable 6. Randomized controlled trials[1] intervening with both vitamin B_6 and B_{12} and reporting on the risk of fracture.

Name of the trial (n)	Intervention	Fractures, n and type	Results, Relative risk (RR) for fracture	Comments
**HOPE-2[2]**<br>(n=5522)	T[a]: 1mg vit B_{12} + 2.5mg folic acid<br>+ 50mg vit B_6<br><br>C[b]: Placebo	N=313<br>(non-vertebral fractures)	**RR=1.07** (0.85,1.33)	Number of hip fractures was not reported.<br>HOPE2 also reported on vertebral fractures (n=50, RR= 0.93 (0.53-1.62) but they are less reliable as many are not clinically recognized.
**VITATOPS[3]**<br>(n=8164)	T: 0.5mg vit B_{12} + 2mg folic acid<br>+ 25mg vit B_6<br><br>C: Placebo	N=145<br>(any fracture)	**RR=0.86** (0.62,1.18)	A lower dose of vit B_6 was used than the other trials.<br><br>70 hip fractures
**WAFACS[4]**<br>(n=4810)	T: 1mg vit B_{12} + 2.5mg folic acid<br>+ 50mg vit B_6<br><br>C: placebo	N=349<br>(nonspine fractures)	**RR=1.08** (0.88–1.34)	Increased risk in post hoc analysis of those with normal/high plasma vit B_6<br>(RR 1.39 (1.01–1.91))<br><br>22 hip fractures
**NORVIT/WENBIT[5]**<br>(n=6837)<br>(factorial design)	T1: 0.4 mg vit B_{12} + 0.8mg folic acid<br>+ 40mg vit B_6<br><br>T2: 0.4 mg vit B_{12} + 0.8mg folic acid<br><br>T3: 40mg vit B_6<br><br>C : placebo	N=236<br>(hip fractures)	T1 (B_6+B_{12}/folic acid) vrs. C:<br>**RR=1.49** (1.05-2.11)<br><br>T2 (B_{12}/folic acid) vrs. C:<br>**RR of 0.89** (0.60-1.32)<br><br>T3 (B_6) vrs. C:<br>**RR of 1.19** (0.83-1.72)	Only hip fractures.<br><br>The results are from an extended follow-up

[a]T=treatment; [b]C=control;

© 2019 Meyer et al. *JAMA Network Open.*

eReferences

1. Garcia Lopez M, Baron JA, Omsland TK, Søgaard AJ, Meyer HE. Homocysteine-Lowering Treatment and the Risk of Fracture: Secondary Analysis of a Randomized Controlled Trial and an Updated Meta-Analysis. *JBMR Plus.* 2018;2:295-303.
2. Sawka AM, Ray JG, Yi Q, Josse RG, Lonn E. Randomized clinical trial of homocysteine level lowering therapy and fractures. *Archives of Internal Medicine.* 2007;167:2136-2139.
3. Gommans J, Yi Q, Eikelboom JW, et al. The effect of homocysteine-lowering with B-vitamins on osteoporotic fractures in patients with cerebrovascular disease: substudy of VITATOPS, a randomised placebo-controlled trial. *BMC geriatrics.* 2013;13:88.
4. Stone KL, Lui L-Y, Christen WG, et al. Effect of Combination Folic Acid, Vitamin B6, and Vitamin B12 Supplementation on Fracture Risk in Women: A Randomized, Controlled Trial. *Journal of Bone and Mineral Research.* 2017;32:2331-2338.
5. Garcia Lopez M, Bønaa KH, Ebbing M, Eriksen EF, Gjesdal CG, Nygård O, Tell GS, Ueland PM, Meyer HE. B Vitamins and Hip Fracture: Secondary Analyses and Extended Follow-Up of Two Large Randomized Controlled Trials. *Journal of Bone and Mineral Research.* 2017;32:1981-1989.

© 2019 Meyer et al. *JAMA Network Open.*
